# A graphitic nano-onion/molybdenum disulfide nanosheet composite as a platform for HPV-associated cancer-detecting DNA biosensors

**DOI:** 10.1186/s12951-023-01948-6

**Published:** 2023-06-10

**Authors:** Youngjun Kim, Eunah Kang

**Affiliations:** School of Chemical Engineering and Material Science, Chung-Ang University, 221 Heukseok-Dong, Dongjak-Gu, Seoul, Republic of Korea

**Keywords:** Nano-onion, HPV, DNA, Electrochemical biosensor, Molybdenum disulfide, Amorphous nanocarbon supports, Nanocomposites

## Abstract

An electrochemical DNA sensor that can detect human papillomavirus (HPV)-16 and HPV-18 for the early diagnosis of cervical cancer was developed by using a graphitic nano-onion/molybdenum disulfide (MoS_2_) nanosheet composite. The electrode surface for probing DNA chemisorption was prepared via chemical conjugation between acyl bonds on the surfaces of functionalized nanoonions and the amine groups on functionalized MoS_2_ nanosheets. The cyclic voltammetry profile of an 1:1 nanoonion/MoS_2_ nanosheet composite electrode had an improved rectangular shape compared to that of an MoS_2_ nanosheet elecrode, thereby indicating the amorphous nature of the nano-onions with sp_2_ distancing curved carbon layers that provide enhanced electronic conductivity, compared to MoS_2_ nanosheet only. The nanoonion/MoS_2_ sensor for the DNA detection of HPV-16 and HPV-18, respectively, was measured at high sensitivity through differential pulse voltammetry (DPV) in the presence of methylene blue (MB) as a redox indicator. The DPV current peak was lowered after probe DNA chemisorption and target DNA hybridization because the hybridized DNA induced less effective MB electrostatic intercalation due to it being double-stranded, resulting in a lower oxidation peak. The nanoonion/MoS_2_ nanosheet composite electrodes attained higher current peaks than the MoS_2_ nanosheet electrode, thereby indicating a greater change in the differential peak probably because the nanoonions enhanced conductive electron transfer. Notably, both of the target DNAs produced from HPV-18 and HPV-16 Siha and Hela cancer cell lines were effectively detected with high specificity. The conductivity of MoS_2_ improved by complexation with nano-onions provides a suitable platform for electrochemical biosensors for the early diagnosis of many ailments in humans.

**Graphical Abstract:**

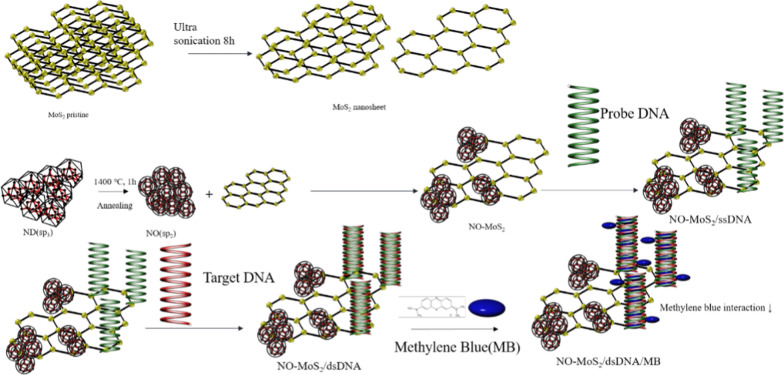

**Supplementary Information:**

The online version contains supplementary material available at 10.1186/s12951-023-01948-6.

## Introduction

Molybdenum disulfide (MoS_2_) capable of forming 2D graphene-like nanosheets has opened up a new research field due to its unique mechanical, electrical, thermal, and optical properties [1]. They are created by stacking S-Mo-S layers via Van der Waals interactions. The easiest approach for obtaining MoS_2_ nanosheets is the top-down method of breaking down bulk MoS_2_ into thin 2D layers via liquid separation or by inserting ions into a weakened intercalating layer. MoS_2_ has attracted a great deal of attention for use in chemical detection and biomolecule sensing platforms as well as optoelectronics, supercapacitors, and batteries [2–5]. MoS_2_ nanosheets have been applied in optochemical biosensors by exploiting MoS_2_ fluorescence quenching of fluorophore-labeled single-strand (ss) DNA to detect protein or DNA [6]. Moreover. MoS_2_ has been developed with hybrid structure to improve electrical conductivity and sensitivity, combining with metals [7], gold nanoparticles [8–10], polyaniline [11], polypyrrole [12], PEDOT [13], carbon materials [14–17], etc., to provide electrochemical sensors with better electrical conductivity and sensitivity at low cost and common scale facility, and avoid having to label DNA.

Versatile carbon nanostructures including nanotubes, graphene oxide, nanodiamonds, etc. have been utilized as immobilization platforms for DNA [18–20]. The electroconductivity of graphene-like carbon materials can be improved by introducing lattice defects [21], increasing the porosity on the substrate [22, 23], and adding doping impurity [24–28]. For these reasons, hybrid MoS_2_/graphene-like sensor surface platforms have been developed with diverse functionality based on fluorescence quenching [5], electrochemistry [7, 16, 29], enzyme protein-mediated electrochemistry [30], calorimetry [6], etc. However, the surface of a DNA sensor requires DNA labeling with a fluorescent dye, the biomolecules must be concentrated beforehand due to low sensitivity, and easy-to-use clinical table-top devices are required.

Nano-onions possess graphitic sp_2_ structures and are derived from crystalline sp_3_ nanodiamonds by thermal annealing or laser irradiation [31, 32]. Their attractive characteristics are easy surface chemical modulation, excellent electron transfer capacity, and good biocompatibility due to an amorphous carbon distancing layer with a hexagonal and pentagonal curved structure and chemical dangling functional group. For these structural flexibility, amorphous nanoonion may allow to adapt easily the surface of MoS_2_ and generating intimate interface between nano-onion and MoS_2_, leading improved electrochemical performance [33].

**Scheme 1:**
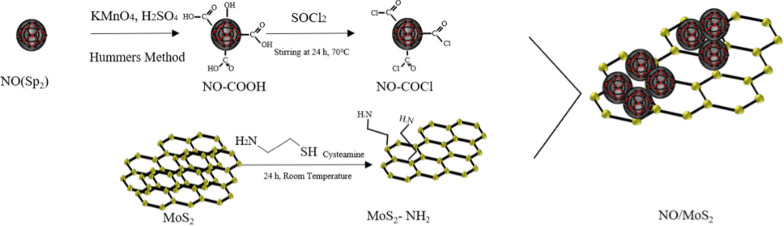
A schematic presentation of the chemical conjugation between the acyl groups on the functionalized nano-onions and the amine groups on the functionalized MoS_2_ nanosheets. KMnO_4_, potassium permanganate; H_2_SO_4_, sulfuric acid; SOCl_2_, thionyl chloride; NO, nano-onion; ss, single-strand; COCl, acyl chloride; NH_2_, amine; SH, thiol

**Scheme 2:**
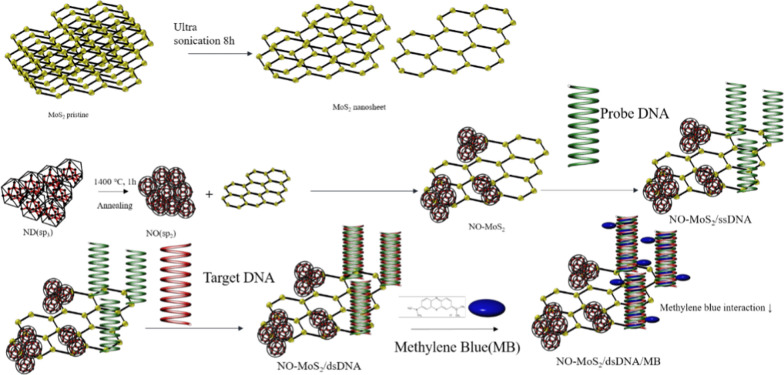
A schematic representation of the DNA-based electrochemical detection of HPV using the nano-onion/MoS_2_ nanosheet composite platform. ND, nanodiamond; NO, nano-onion; ss, single-strand

Hence, a new hybrid comprising nano-onions and MoS_2_ nanosheets could overcome the problem of low conductivity of the MoS_2_ nanosheets in a biosensor surface platform. In this study, nano-onion/MoS_2_ nanosheet composites were synthesized via surface oxidation and acylation of the nano-onion surface to enable chemical conjugation with MoS_2_ nanosheets, while the amine-modified MoS_2_ surface enabled chemical conjugation with the nano-onions and DNA chemical adsorption (Scheme 1). The nano-onion /MoS_2_ biosensor platform with immobilized unlabeled ssDNA was prepared to detect target cervical cancer-expressing DNA from human papillomavirus (HPV)-16 and HPV-18 DNA that was chosen for the early diagnosis of cervical cancer. Unlabeled HPV ssDNA was chemically conjugated onto the NO/MoS_2_ surface, while methylene blue (MB) employed as a redox electronic indicator was intercalated with hybridized double-stranded (ds) DNA (Scheme 2). Thereby, enhanced surface platform with the sensitivity was characterized by using differential pulse voltammetry (DPV) with low limit of detection [34]. Early sensitive detection by using unlabeled ssDNA-based electrochemical biosensors could provide early clinical diagnoses for a variety of diseases.

## Results and discussion

**Fig. 1:**
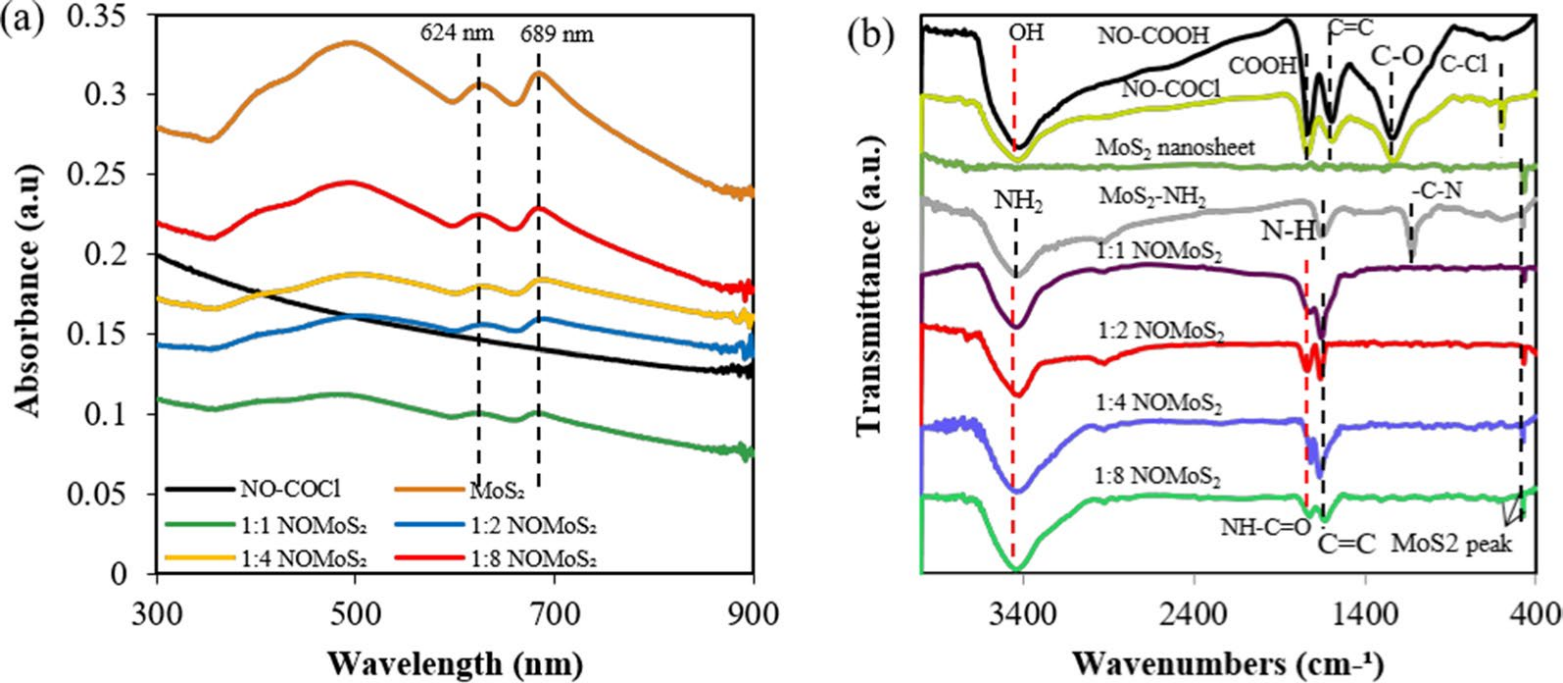
**a** UV–vis absorbance spectra and **b** FT-IR spectra of nano-onions, MoS_2_ nanosheets, and nano-onion/MoS_2_ nanosheet composites with ratios from 1:1 to 1:8. NO, nano-onion; COCl, acyl; NH_2_, amine; COOH, carboxyl

The optical properties of MoS_2_ nanosheets and nano-onion/MoS_2_ nanosheet composites with various weight ratios (1:1–1:8) were measured using UV–vis absorbance spectra (Fig. 1a). The spectra of MoS_2_ nanosheets showed excitonic absorption peaks at 624 and 689 nm, which originated from the direct gap transitions at the K point of the Brillouin zone and were induced by energy splitting of the valence band and spin orbital coupling. An additional peak at around 460 nm is attributed to the optical transition between the density of states peak in the valence and conduction bands [35]. The first peak in the longer wavelength region corresponds to the lowest optical band gap of ~ 1.8 eV of the MoS_2_ nanosheets [36], which is higher than that of bulk MoS_2_ (~ 1.2 eV) [37]. This difference indicated quantum confinement in the nanosheets and their optical characteristics were well preserved in the nano-onion/MoS_2_ nanosheet composites.

FT-IR spectroscopy was used to identify functional groups in the samples (Fig. 1b). The FT-IR spectra of nano-onion-COOH and nano-onion-acyl chloride show strong absorption bands at 3,400 cm^−1^ (hydroxyl bond stretching) and at 1,625 cm^−1^ (C = C bond bending). A peak at 593 cm^−1^ was attributed to acyl chloride bond (C–Cl) stretching in nano-onion-acyl chloride, whereas it was not present in nano-onion-COOH. The FT-IR spectra of nano-onion-acyl chloride, aminated MoS_2_ nanosheets, and nano-onion/MoS_2_ nanosheet composites were compared to verify the conjugation of the amide bonds (NH–C = O) between nano-onion-acyl chloride and the amine-functionalized MoS_2_ nanosheets (N − H deformation vibration peak at 1604 cm^−1^ and an NH_2_ stretching peak at 3390 cm^−1^). The nano-onion/MoS_2_ nanosheet composites displayed the characteristics of both the nano-onions and MoS_2_ nanosheets with the disappearance of the C–Cl stretching peak at 593 cm^−1^. The composites exhibited absorption bands attributed to hydroxyl bond stretching at 3400 cm^−1^. The peak at 1632 cm^−1^, corresponding to the NH-C = O group in the nano-onion/MoS_2_ nanosheet composites, was attributed to overlapping C = C vibrations and C = O stretching, signifying successful chemical conjugation between the materials via amide bonds. Overall, results demonstrated the preservation of optical properties in NO/MoS_2_ nanosheet composites and their successful chemical conjugation.

**Fig. 2:**
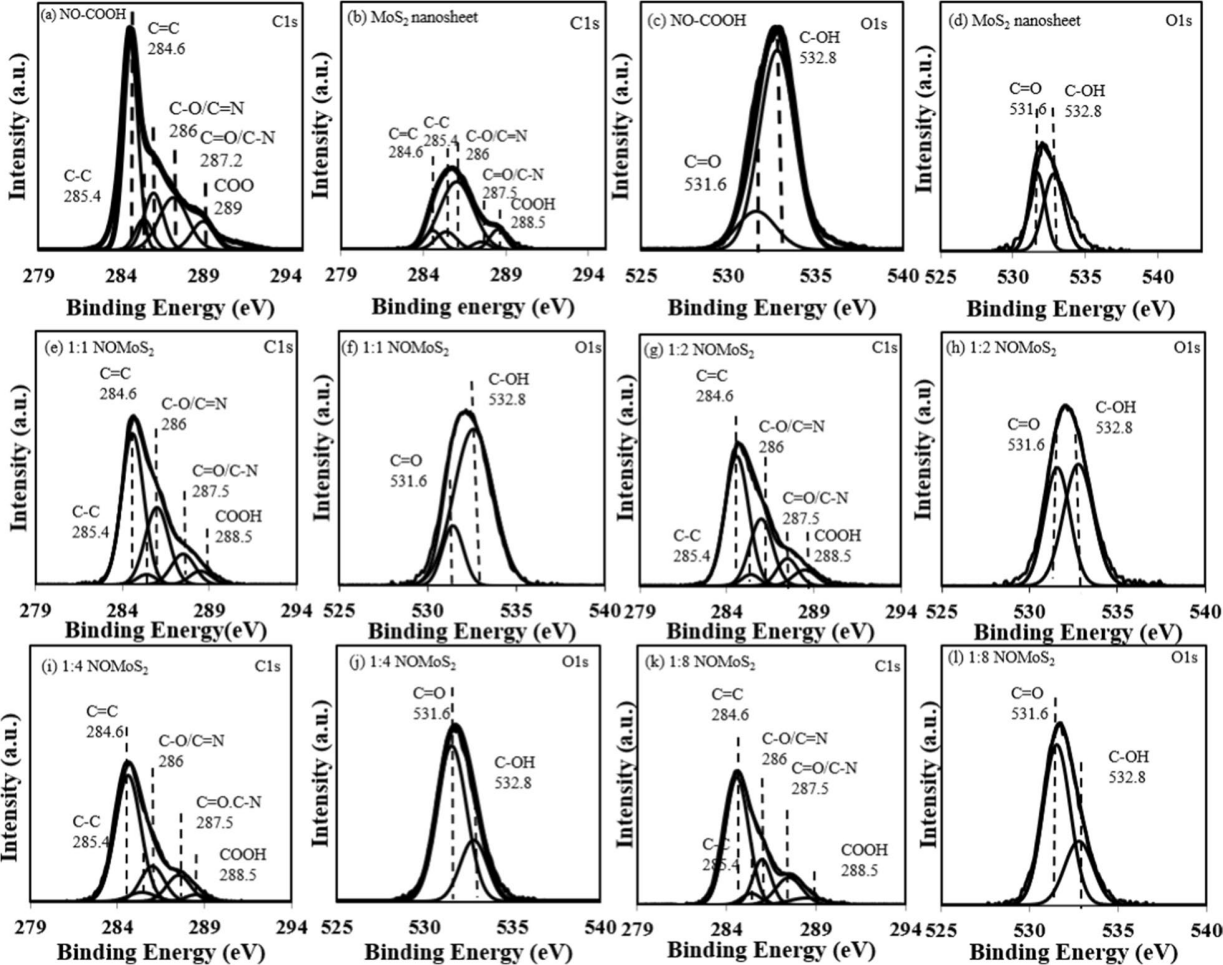
Deconvoluted XPS spectra of nano-onion (NO)-COOH, MOS_2_ nanosheets, and NO/MoS_2_ nanosheet composites. C 1 s spectra of **a** NO-COOH and **b** MoS_2_ nanosheets. O 1 s spectra of **c** NO-COOH and **d** MoS_2_ nanosheets. C 1 s spectra of NO/MoS_2_ nanosheet composites with weight ratios of **e** 1:1, **g** 1:2, **i** 1:4, and (k) 1:8. O 1 s spectra of NO/MoS_2_ nanosheet composites with weight ratios of **f** 1:1, **h** 1:2, **J** 1:4, and **l** 1:8

The chemical structures of nano-onion-COOH, MoS_2_ nanosheets, and nano-onion/MoS_2_ nanosheet composites were investigated by using XPS, the results of which are shown in Fig. 2. The C 1s spectra were deconvoluted to provide evidence for the formation of the nano-onion/MoS_2_ nanosheet composites [38]. Nano-onions derived from nanodiamonds have an unsaturated vinyl C 1 peak at 284.6 eV (C = C), indicating that onion-like amorphous carbon was generated during thermal annealing (Fig. 2a). Surface oxidation of the nano-onions treated by using Hummer’s method was confirmed by the strong oxidation peak intensities at 287.2 eV (C–O) and 289 eV (COOH). High oxygen content is indicated by the carbonyl and carboxyl peaks at 531.6 and 532.8 eV (Fig. 2c), thereby proving that the oxygen-terminated surface functional groups were generated on the outer nano-onion shells, which could be chemically conjugated with the MoS_2_ nanosheets. Nano-onion-COOH has a high chemical composition of C = C sp_2_ bonds (approx. 45.1%), while the MoS_2_ nanosheets showed weak peak attribution.

The XPS C 1s spectra of the nano-onion/MoS_2_ nanosheet composites in Fig. 2e indicate that the C = C peak became smaller as the amount of MoS_2_ nanosheets was increased, which indicates the formation of a composite comprising nano-onions and MoS_2_ nanosheets. In addition, the C = O/C–N ratio of the nano-onion/MoS_2_ nanosheet composites increased as the amount of MoS_2_ nanosheets was increased, with ratios of 1:2, 1:4, and 1:8 achieving 11.2%, 9.1%, and 14.2%, respectively. And C = O/C–N peak position of the nano-onion/MoS_2_ nanosheet composites was accompanied by a shift to a higher energy binding energy (from 287.2 to 287.5 eV). Semi-electron-rich MoS_2_ nanosheets on the nano-onions facilitate electron transfer into the nano-onion graphitic shell layer, resulting in increased electron mobility between the nano-onions and MoS_2_ nanosheets. The C = O/C-N composition of nano-onion-COOH and MoS_2_ nanosheets were 21.8%, and 3.9%, respectively. The deconvoluted O 1s spectra of the nano-onions and nano-onion/MoS_2_ nanosheet composites show peaks at 531.6 eV for C = O and 532.8 eV for C–OH. As the amount of MoS_2_ nanosheets was increased, the peak intensity of C–OH at 532.8 eV lessened, indicating that the carboxylic groups in the nano-onions had chemically conjugated with the MoS_2_ nanosheets.

**Fig. 3:**
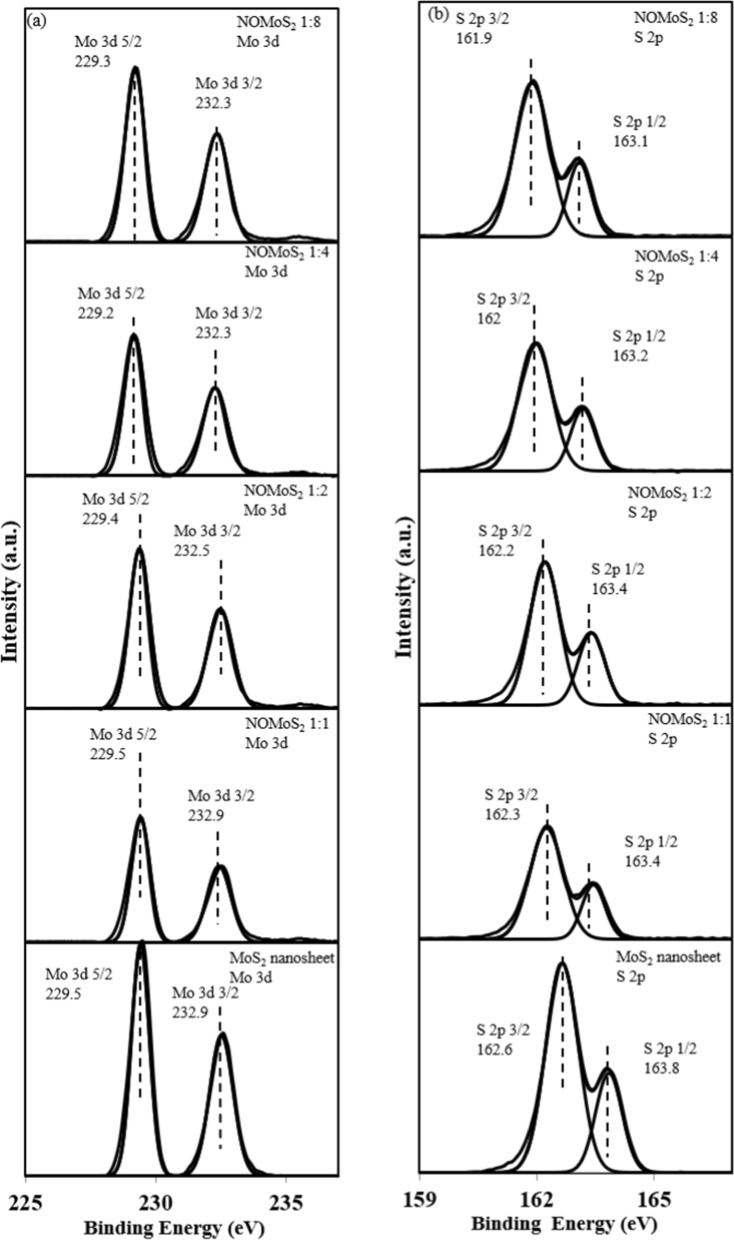
Deconvoluted XPS **a** Mo 3d spectra and **b** S 2p spectra for the MoS_2_ nanosheets and nano-onion/MoS_2_ nanosheet composites with ratios from 1:1 to 1:8

The Mo and S chemical composition and bonding states in the MoS_2_ nanosheets and nano-onion/MoS_2_ nanosheet composites were investigated by using appropriate deconvoluted XPS spectra (Fig. 3). The deconvoluted XPS Mo 3d spectra show two dominant peaks and a smaller peak at a higher binding energy for both the MoS_2_ nanosheets and nano-onion/MoS_2_ nanosheet composites [39]. In the nano-onion/MoS_2_ nanosheet composites, the chemical composition of Mo 3d was relatively strengthened as the amount of MoS_2_ nanosheets was increased. Moreover, the peaks were slightly shifted to lower binding energies in the nano-onion/MoS_2_ nanosheet composites, compared to the MoS_2_ nanosheets. This infers that complexation of the nano-onions and MoS_2_ nanosheets occurred and resulted in a small rise in the electron density around Mo in the nanocomposites, a phenomenon that is similar to N-doped MoS_2_ [40]. The deconvoluted XPS S 2p spectra in Fig. 3b provide the same results as for Mo 3d with the highest composition ratio in the MoS_2_ nanosheet and the lowest in the 1:1 nano-onion/MoS_2_ nanosheet composite. The S 2p doublet peaks were assigned as S 2p_1/2_ at 163.8 and S 2p_3/2_ at 162.6 eV, that is attributed to the binding energy of divalent sulfide (S^2−^) ions in stoichiometric MoS_2_ [41, 42]. Downshift of S 2p binding energy and decrease of binding energy indicate that sulfur may be reduced than the valance state of divalent sulfide (S^2−^), providing election-rich S chemical environment [43]. Similar to the Mo 3d spectra of composite, shifting of the peaks to lower binding energies in the nano-onion/MoS_2_ nanosheet composites indicates that nano-onion complexation with MoS_2_ nanosheets provided higher electron densities around the Mo and S atoms.

**Fig. 4:**
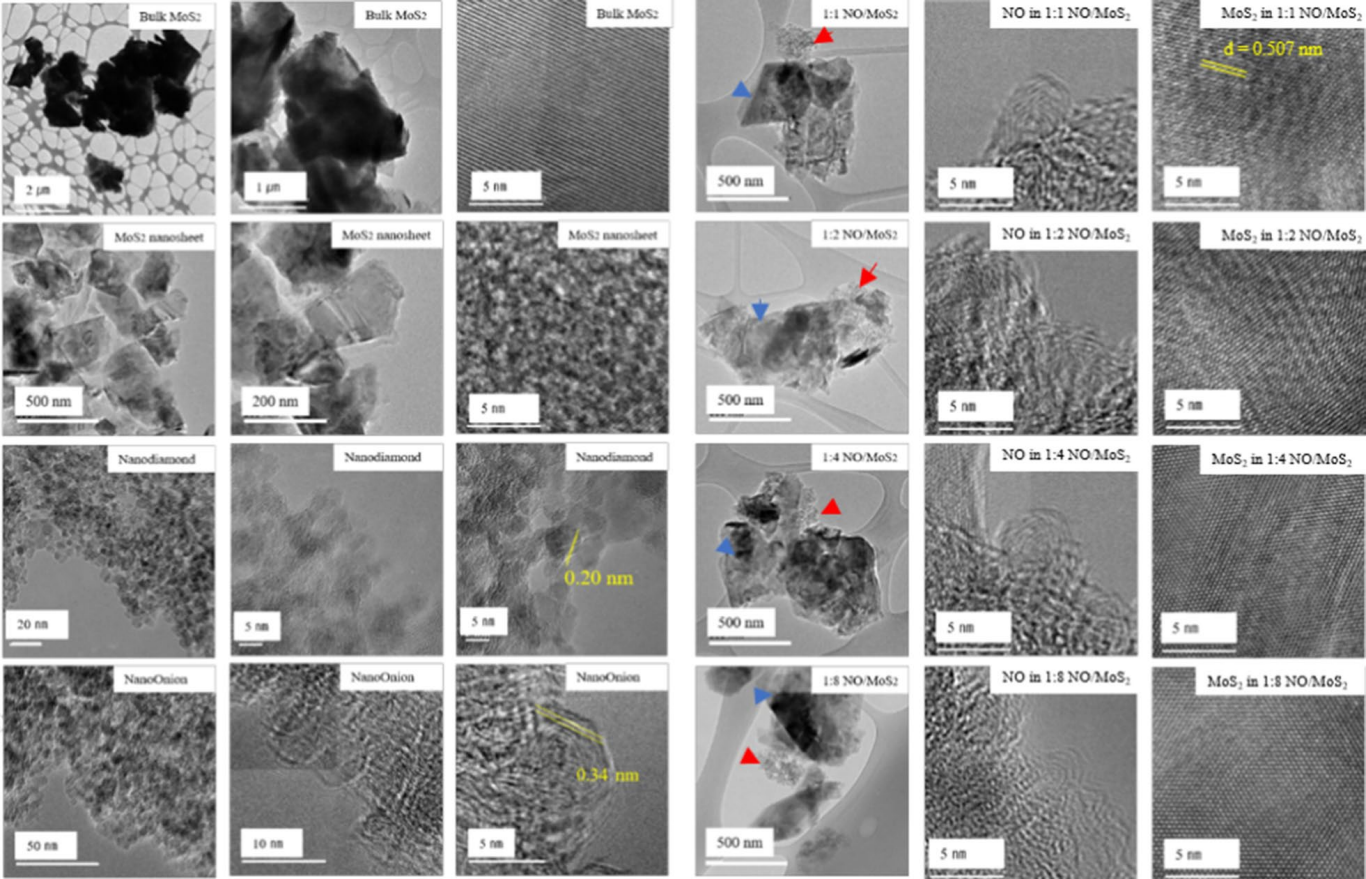
HRTEM images of bulk MoS_2_, MoS_2_ nanosheets, nanodiamonds, nano-onions, and nano-onion (NO)/MoS_2_ nanosheet composites with weight ratios from 1:1 to 1:8. Images of the forth column present nano-onion (NO)/MoS_2_ nanosheet composites with ratio. The fifth column and the sixth column present, respectively, the magnified images of NO and MoS_2_ nanosheet on the fringe of the nano-onion (NO)/MoS_2_ nanosheet composite from the image of fourth column. The red and blue arrows indicate the agglomerate NO and MoS_2_ nanosheets in composite form, respectively

Structural visualization of bulk MoS_2_, MoS_2_ nanosheets, nanodiamonds, nano-onions, and nano-onion/MoS_2_ nanosheet composites are revealed in the HRTEM images in Fig. 4. The MoS_2_ nanosheets were quite transparent, indicating their nanoscale thickness. The lateral size of the MoS_2_ nanosheet ranged from 400 to 500 nm. Typical TEM images of MoS_2_ nano sheet was displayed, which can be indexed to crystal plane of (002), (100), (103), (105) of hexagonal MoS_2_. And its stripe-like grain lattice on the edge of MoS_2_ nanosheet at high magnification displayed interlayer distance between fringes to be 0.507 nm, which can be indexed to *d*-space of (002) crystal plane. The peak 2θ = 14.4^o^ was also displayed from the XRD spectra (Additional file 1: Fig. S1). Using Bragg equation, the lattice spacing (*d*) are calibrated to be 6.87 Å, which present related previous results [44].

The onion-like curved layer shown in the HRTEM images of nano-onions verified the sp_2_ structure of the carbon atoms in both hexagonal and pentagonal arrangements, which is unlike planar graphene. The curved layer in the nano-onions resulted in a greater lattice spacing in their molecular layer (0.34 nm) than in nanodiamonds (0.2 nm), as coincidence with previous study [45, 46]. In the nano-onion/MoS_2_ nanosheet composites with ratios from 1:1 to 1:8, agglomerates of nano-onions were well enveloped in the MoS_2_ nanosheets, thereby indicating their stable binding together.

**Fig. 5:**
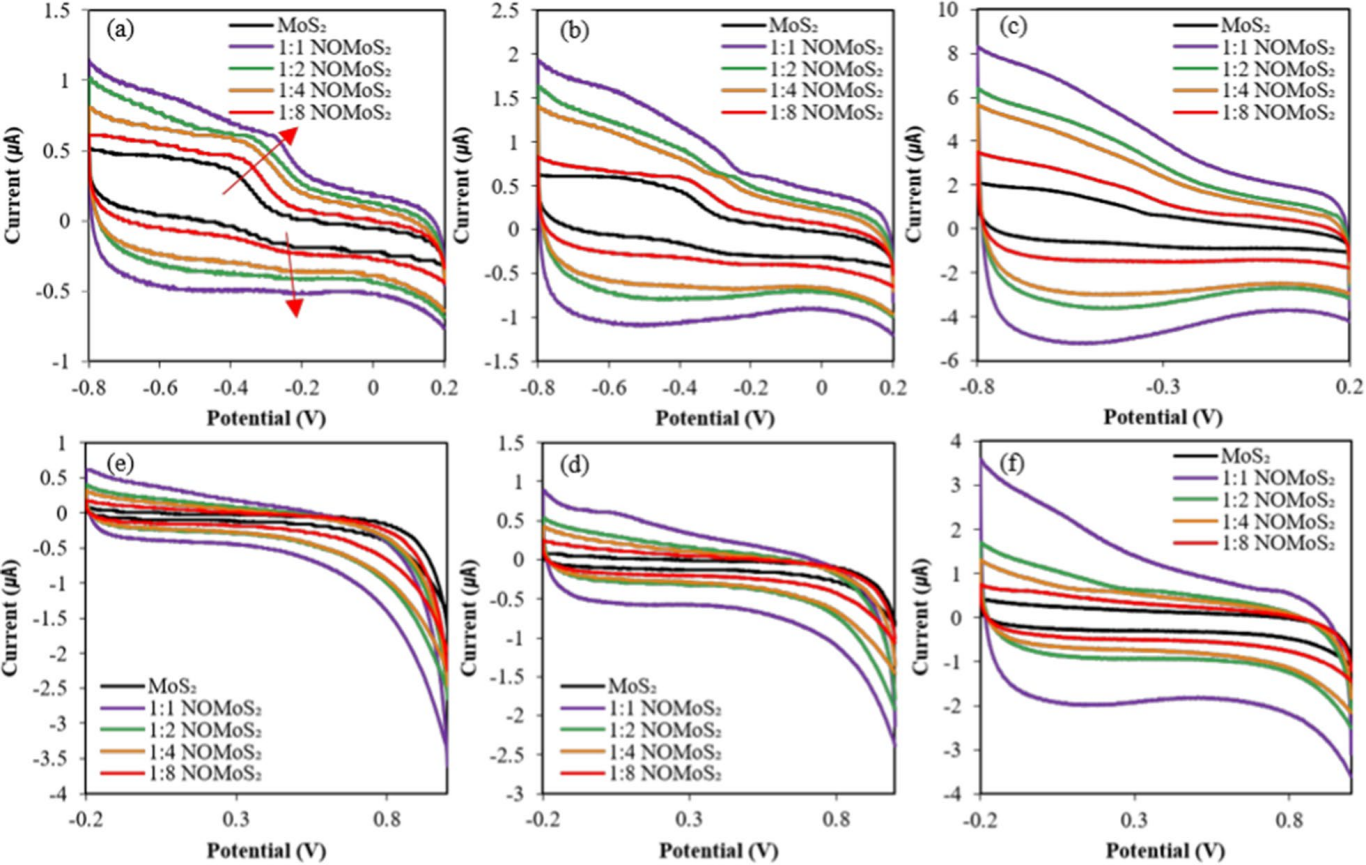
CV profiles of MoS_2_ nanosheets and nano-onion/MoS_2_ nanosheet composites with ratios of 1:1, 1:2, 1:4, and 1:8. The potential ranges of first **a**, **b**, **c** and second row **d**, **e**, **f** were -0.8 to 0.2 V and -0.2 to 1.0 V, respectively. The scan rate of the left **a**, **d**, mid **b**, **e** and right **c**, **f** column was, respectively as 0.05, 0.1, or 0.5 V/s. NO, nano-onion

CV profiles of electrodes, comprising MoS_2_ nanosheets or nano-onion/MoS_2_ nanosheet composites with weight ratios from 1:1 to 1:8, were obtained at scan rates of 0.05, 0.1, and 0.5 V/s in 0.1 M potassium hydroxide (KOH). (Fig. 5). The MoS_2_ nanosheets shows the quasi- rectangular CV profile and the small shoulder of redox peak, indicating a double-layer reaction and pseudocapacitive behavior with a fast-reversible redox reaction, respectively, as previously reported [47–49]. The peak shoulder may be due to redox reaction of Mo active site on the composite edge, as displayed with red arrow in Fig. 5a. The CV curves of MoS_2_ nanosheets at 0.05 V/s in the potential range from -0.8 to 0.2 V (Fig. 5a) suggest high electrochemical stability at the active sites in MoS_2_ and good reversibility due to fast electrolyte ion diffusion. The 1:1 nano-onion/MoS_2_ nanosheet composite electrode exhibited a significantly improved current density compared to the MoS_2_ nanosheet electrode, by attribution of nano-onion presence. The enhanced current density of 1:1 nano-onions/MoS_2_ nanosheeet composite electrode resulted from high conductivity and effectiveness of MoS_2_ active sites facilitated by intercalation and deintercalation of K^+^ ion during redox process [50].

Two possible redox reaction mechanism of surface could be suggested from peaks in the CV profiles of MoS_2_-based electrodes (Eqs. 1 and 2) [50–52]. The non-faradaic process, due to double layer formation, may occur during the adsorption of cation on the MoS_2_ nanosheet on the electrode (Eq. 1).

1 $$\left( {{\text{MoS}}_{{2}} } \right)_{{{\text{Surface}}}} \, + \,{\text{K}}^{ + } \, + \,{\text{e}}^{ - } \, \leftrightarrow \,\left( {{\text{MoS}}_{{2}} \, - \,{\text{K}}^{ + } } \right)_{{{\text{Surface}}}}$$

The pseudo-capacitive behavior of CV profile suggests that alkali cation (K^+^) diffuse in the interlayer of MoS_2_ nanosheet with nanoonion interface (Eq. 2).

2 $$\tt {\text{MoS}}_{{2}} \, + \,{\text{xe}}^{ - } \ + \,{\text{xK}}^{ + } \, \leftrightarrow \,({\text{MoS}}{-}{\text{SK}}^{ + } )$$

The improved current density of the nano-onion/MoS_2_ nanosheet composite electrodes could be due to better facilitation of electron transfer. The 1:1 nano-onion/MoS_2_ nanosheet composite exhibited the highest current density, while other composites showed lower current densities but higher than the MoS_2_ nanosheet electrode. This could be ascribed to the fact that the adsorption and desorption processes of electrolyte ions (K^+^) on the surface and MoS_2_ intra/interlayers could be affected by the electron transfer capability of the nano-onions.

CV curves performed at different scan rate demonstrated that NO complex formation provides effective intercalation of K^+^ ion by generating additional conductive paths, enhancing charge storage, and providing additional oxidation and reduction pathways. Nano-onion composite with MoS_2_ nanosheet would facilitate heterogeneous charge transfer, ion diffusion and capacity by providing platform of surface defect and active surface sites. Nano-onion may contribute to the distributed Mo active site and edge, resulting in enhanced distance of ion diffusion and facilitated electron transfer capability [47, 50, 53], and also play the dual roles of electronic transition and a conductive electron donor. The nano-onion/MoS_2_ nanosheet composites facilitated electron transfer at the interface and enhanced the electrochemical functionality of the electrode. The 1:1 nano-onion/MoS_2_ nanosheet composite which overcomes the low conductivity of MoS2 nanosheets was chosen as the DNA linking platform substrate.

DNA hybridization with a DNA probe and target DNA (HPV-16 or HPV-18) was investigated using the 1:1 nano-onion/MoS_2_ surface platform. Methylene blue (MB) served as a redox indicator for electron transfer reactions, intercalating with dsDNA via electrostatic adsorption for target DNA detection. DNA hybridization and MB intercalation was performed at the DNA concentration of 5 ng/ml at reaction time of 10 min [11, 54]. The redox reaction of MB transfers one hydrogen ion and two electrons to the elongated and rigid hybridized dsDNA with the aid of a planar aromatic ring, thereby making it possible to examine the current charge via highly sensitive DPV with low limit of detection [55]. The current peaks via DPV appeared at ~ 1.1 V on the MoS_2_ nanosheet and 1:1 nano-onion/MoS_2_ electrodes.

**Fig. 6:**
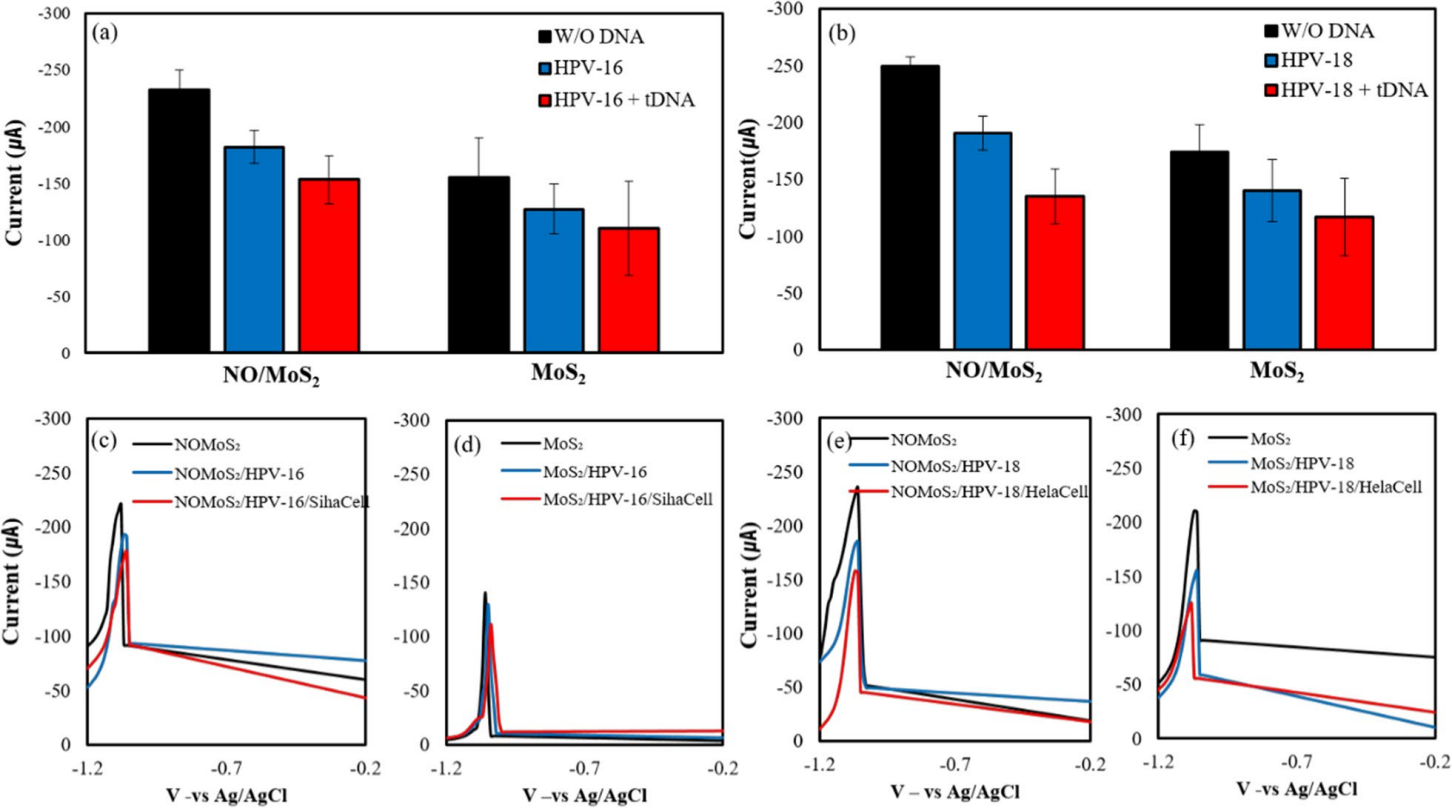
DPV profiles of the 1:1 nano-onion (NO)/MoS_2_ nanosheet composite and MoS_2_ nanosheet electrodes obtained with 1 µM methylene blue (MB; pH 7.0) in potassium chloride (KCl) in 1 ml volume flow cell. Figure **a**, **c** and **d** and Figure **b**, **e**, and **f** displays the sensing potential for HPV-16 and HPV-18, respectively. Figure **a** and **b** display average peak current calibrated from independent measurement of five distinct electrodes. Figure **c** through **f** showcase the DPV profile of a single electrode in sequential mode: without DNA, following probe DNA chemisorption, and after target DNA hybridization (5 ng/ml) for both NO/MoS_2_ nanosheet composite and MoS_2_ nanosheet electrodes

Figure 6 shows DPV profiles for the MB electrochemical oxidation on the 1:1 nano-onion/MoS_2_ nanosheet composite and MoS_2_ nanosheet electrodes. Functional groups on the electrode surface were chemically conjugated with the HPV target probe, followed by sequential DPV measurements. The chemisorption of the HPV-16 and HPV-18 target DNA probes was sensed in HPV-16-positive Siha cells (Fig. 6a, c, d) and HPV-18-positive Hela cells (Fig. 6b, e, f), respectively. The average value of DPV current peaks measured from each electrodes were shown for NO/MoS_2_ nanosheet composite and MoS_2_ nanosheet electrode thought DNA adsorption and HPV target DNA hybridization, sequentially, under exposure of electrolytes with 1 μM MB and 0.1 M KCl (pH 7.0). Figure 6c–f present reduction peak in continuous and subsequent detection manner of after target DNA chemisorption (blue line) and after target DNA hybridization (red line) on each electrode.

The 1:1 nano-onion/MoS_2_ nanosheet composite electrode attained a higher current peak than the MoS_2_ nanosheet electrode, thereby confirming the synergistic effect of the nano-onions combined with MoS_2_ nanosheets. The differential current peaks obtained with both electrodes indicate the occurrence of the MB redox reaction at the electrode-buffer interface and probe.

The catalytic properties of nano-onions and the hexagonal and pentagonal graphitic onion-like layer facilitate excellent electron transfer and conductivity to the nano-onion/MoS_2_ nanosheet composite electrode [21, 38, 56]. The thiolated DNA probes for HPV-16 and HPV-18 were chemisorbed on both the MoS_2_ nanosheet and 1:1 nano-onion/MoS_2_ nanosheet composite electrodes. The conductive carboxylated nano-onions and amine-functionalized MoS_2_ nanosheets enable the electrode surface to chemisorb the DNA probe, reducing the DPV current peak. Notably, the DPV current peak of the DNA probe on the 1:1 nano-onion/MoS_2_ nanosheet composite electrode was higher than that of the MoS_2_ nanosheet electrode throughout all sequential process of DNA detection. The addition of Siha and Hela positive HPV target ssDNA fragments lowered the DPV current peak, indicating successful hybridization of oligonucleotides with the capture probes. MB intercalation into the hybridized DNA did not effectively elevate the current peak because other DNA fragments from the Siha and Hela cells could hybridize with MB, thereby reducing the accessibility of the guanine bases in the dsDNA on the surface [57]. A larger reduction in the DPV current peak for MB was induced by target DNA detection, confirming greater amplification of the differential sensing signal for both HPV cell lines with the 1:1 nano-onion/MoS_2_ nanosheet composite electrode, compared to the MoS_2_ electrode. The excellent electronic conductivity and chemical functionalization of the 1:1 nano-onion/MoS_2_ nanosheet composite makes it potentially applicable to a wide variety of biological molecules.

**Fig. 7:**
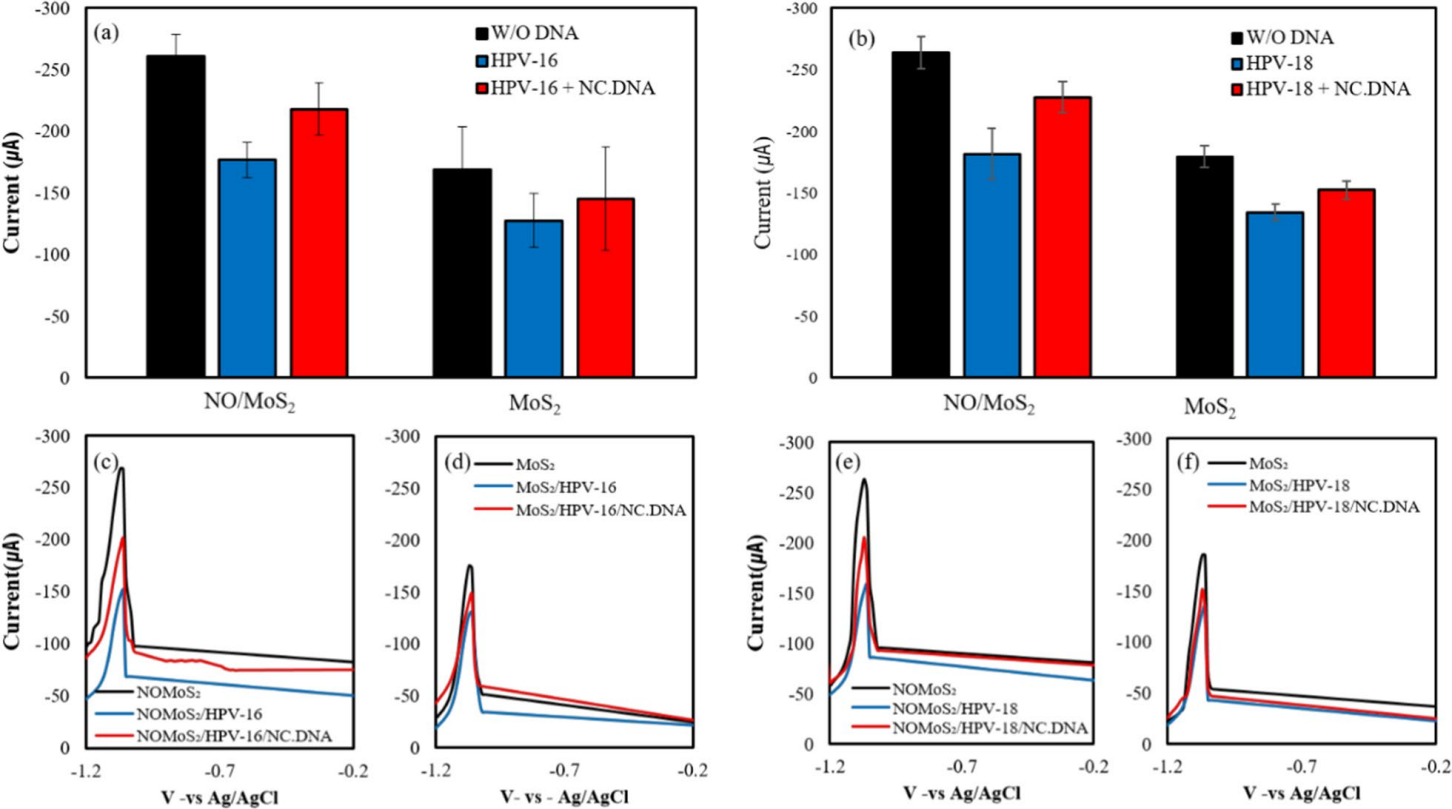
DPV profiles of ssDNA/nano-onion (NO)/MoS_2_/indium tin oxide (ITO) and ssDNA/MoS_2_/ITO + non-complementary (NC) DNA (5 ng/ml). Figure **a**, **c** and **d** and Figure **b**, **e**, and **f** displays the sensing potential for HPV-16 and HPV-18, respectively. Figure **a** and **b** display average peak current calibrated from independent measurement of five distinct electrodes. Figure **c** through **f** showcase the DPV profile of a single electrode in sequential mode: without DNA, following probe DNA chemisorption, and after addition of noncomplimentary DNA for both NO/MoS_2_ nanosheet composite and MoS_2_ nanosheet electrodes

To confirm the specificity of the DNA targeting by the sensor, non-complementary DNA was added to the HPV16 and HPV18 probe DNA surface electrodes, and then the current change by DPV was measured on 1:1 nano-onion/MoS_2_ nanosheet composite and MoS_2_ electrodes. Figure 7a, b display average peak current for five different electrode for HPV-16 and HPV-18 detection, respectively. Figure 7a, b also show the current variation that occur when non-complementary DNA (5 ng/ml) is introduced to the sensor probes, specifically designed for detecting HPV-16 and HPV-18, respectively. Figure 7c throughout Fig. 7f displays DPV profile in a continuous and sequential detection approach after probe DNA chemisorption on electrode surface (blue line) and following non-complimentarty DNA (red lien) for both NO/MoS_2_ nanosheet composite and MoS_2_ nanosheet electrodes. Upon adding non-complimentary DNA, an increase in DPV peak current amplitude was observed for both HPV-16 and HPV-18. The prominent enhancement in the DPV peak current signal could be ascribed to the electrical coupling between the groove in ss DNA attributable to guanine and MB. When the non-complementary DNA was added to the sensor chamber, the DPV current peak increased, allowing MB to intercalate into the groove of ssDNA, due to the absence of double helix formation. Higher current peak value upon exposure of NC DNA and MB, compared to that of DNA probe electrode could potentially stem from NC DNA/MB interaction and subsequent nonspecific absorption. Dynamic interaction between NC DNA and MB in the electrolytes solution may be elevated to enhance electron transfer on the DNA probe electrode surface because MB in the electrolytes may play a role as back filling agent in the DNA probe electrode surface [58]. The distinct current peak value of DPV under exposure NC DNA can be attributed to the fact that HPV-16 and HPV-18 positive DNA probe electrode has a specific affinity for complimentary DNA wherein MB is intercalated within the double strand.

**Fig. 8:**
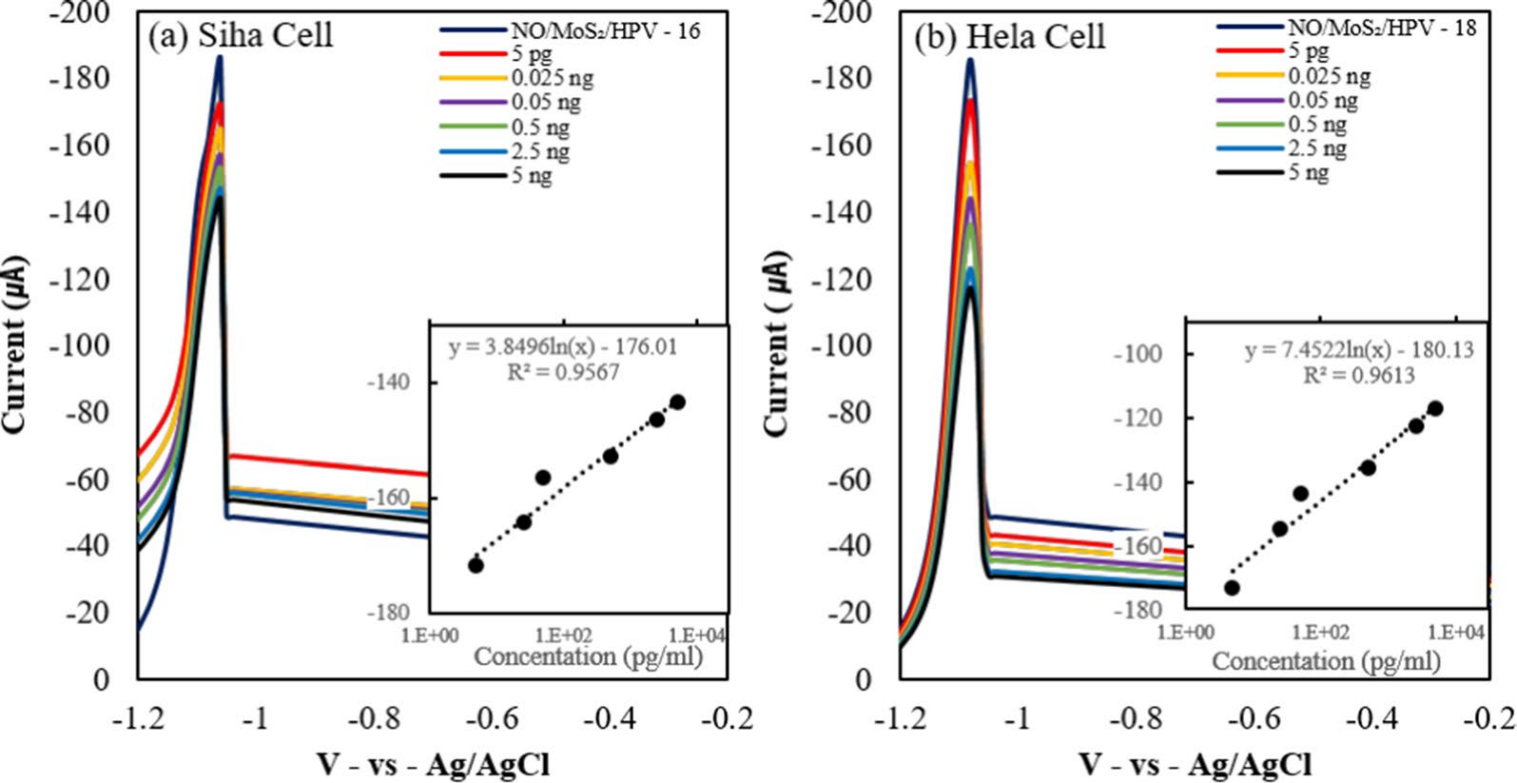
DPV profiles for the detection of various concentrations of target DNA ranging from 5 pg to 5 ng by the 1:1 nano-onion (NO)/MoS_2_ nanosheet composite sensor. **a** HPV-16 in Siha cells and **b** HPV-18 in Hela cells. The graphs in the inlet box shows linear regression of peak at each concentration

DPV responses were recorded after the addition of target DNA at various concentrations ranging from 5 pg to 5 ng with 1 µM MB in KCl (pH 7.0) (Fig. 8). Inlet box within the figure presents the linearity of linear regression, ranging from 5 pg to 5 ng/ml. The anodic peak current in presence of MB decreased after the target DNA was increased. The results indicate that the intercalation of MB between dsDNA bases was restricted and thus resulted in reduced oxidation due to steric hindrance of the bonded bases. For the HPV-16 probe DNA in the 1:1 nano-onion (NO)/MoS_2_ nanosheet composite sensor (Fig. 8a), the increased concentration of HPV-16 positive DNA in Siha cells decreased the anodic peak current due to the more restricted intercalation of MB. Similar results for detecting HPV-18 positive DNA in Hela cells (Fig. 8b) also show that the anodic peak current decreases as the concentration of target DNA increases. Both DPV profiles with varied concentration showed linear relationships with a significant number of R^2^ value (0.956, 0.961), suggesting reliable performance of sensor systems.

The development of electrochemical DNA sensor for HPV detection performed with advancement in recent year, with various surface platform and distinct molecular strategies being employed Table 1. compares the performance of HPV anti-DNA sensors with respect to their limits of detection and linear ranges. Chekin et al. employed a nucleic aptamer-modified porous reduced graphene oxide (prGO)/MoS_2_-based electrode for HPV detection, reporting high sensitivity and selectivity due to the synergistic effect of prGO and MoS_2_ materials and specific binding of the nucleic aptamer to the HPV target [29]. Kowalczyk and Nowicka proposed a voltammetric detection method for HPV 18 using mercury-mediated thymine-base pairs, enabling sensitive and selective detection of HPV 18 [59]. Meanwhile, alternative molecular strategiy by Liu et al. developed a highly sensitive homogeneous electrochemical biosensor based on the CRISPR-Cas12a system, achieving an ultra-low limit of detection (LOD) of 3.21 pM [60]. The limits of detection (LOD) achieved in these studies range from 0.05 fM to 18 nM, highlighting the potential of these biosensors for accurate and sensitive HPV detection. Our study for both HPV-16 and HPV-18 also achieved the LOD of 5 pg/ml at the linear range of 5 pg/ml–5 ng/ml with comparable sensitivity, suggesting reliable diagnostic tools and monitoring for HPV associated diseases.

**Table 1:** Electrochemical HPV sensing platform measured by differential pulse voltammetry

Interface	Analyte	Strategy	LOD	Linear range	Refs
Au/L-cystein	HPV-16	HPV-16 DNA	18.13 nM	18.7–250 nM	[61]
prGO-MoS_2_	HPV-16 L1	RNA L1 aptamer	1.75 pM	3.5–35.3 pM	[29]
GCE	HPV-18	Hg^2+^-thymine	12 fM	–	[59]
NO/MoS_2_	HPV-16, HPV-18	HPV-16 DNA/*MB* HPV-18 DNA/*MB*	5 pg/ml 5 pg/ml	5 pg/ml-5 ng/ml	This work
ITO	HPV-16, HPV-18	HPV-induced CRISPR-Cas12a signal amplification	3.22 pM	0.01–100 nM	[60]
SPCE/rGO,MWCNT, Au nanoparticle, L-cysteine	HPV-18	HPV-18 DNA/*AQMS* anthraquninone-2-sulfonic acid monohydrate sodium salt	0.05 fM	0.01fM-0.01 nM	[54]

Screen-printed carbon electrode (SPCE)

## Conclusions

An electrochemical DNA sensor for detecting HPV-16 and HPV-18 was successfully prepared using a nano-onion/MoS_2_ nanosheet composite. Acyl bonds terminated surface of sp_2_ amorphous graphitic nano-onions derived from nanodiamond formed a composite with amine-functionalized MoS_2_ nanosheets, thereby taking advantage of the synergism with the two material hybrid. As confirmed through DPV, the nano-onion/MoS_2_ nanosheet composite sensor showed high sensitivity due to the amplification of the low electrical conductivity of MoS_2_ by complexation with the nano-onions. Sequential chemical functionalization of the nano-onions and MoS_2_ nanosheets was confirmed via FT-IR and XPS analyses showing characteristic C = C bonding in the sp_2_ structure of the nano-onions and nano-onion/MoS_2_ nanosheet composites. The best CV performance was shown by the 1:1 nano-onion/MoS_2_ nanosheet composite. DPV measurements confirmed that the target DNA in HPV-positive Siha and Hela cells was hybridized with the probe DNA on the electrode surface using MB as a redox indicator. The DPV current peak was lowered after the probe DNA and the target DNA had hybridized. As MB can become electrostatically coupled to guanine in ssDNA, hybridization that form dsDNA made MB intercalation with DNA less effective. As dsDNA is formed, MB is less intercalated into the groove between the electrostatic base and the structure, resulting in a lower oxidation peak. The nano-onion/MoS_2_ nanosheet composite electrode provided a lower DPV current peak than that of the MoS_2_ nanosheet electrode, thereby indicating a more sensitive differential peak probably because of the enhancement of conductive electron transfer by incorporating nano-onions. Notably, both target DNAs produced from HPV-18-positive Siha and HPV-16-positive Hela cancer cells showed good detectability, specificity, and selectivity. Complexation between the nano-onions and MoS_2_ nanosheets improved the conductivity of the latter, thereby providing a biosensor platform for the early diagnosis of a wide variety of human diseases.

## Materials and methods

### Preparation of nano-onion/MoS_2_ Composites and MoS_2_ nanosheets

Graphitized nano-onions were oxidized by using Hummers’ method to generate carboxylic groups on the outer surface [62]. A mixture of sulfuric acid (H_2_SO_4_) (360 mL) and phosphoric acid (H_3_PO_4_) (40 mL) was added to a round-bottomed flask in an ice bath, after which potassium permanganate (KMnO_4_) powder (9 g) was added into cold H_2_SO_4_/H_3_PO_4_ mixture. Nano-onions (3 g) were also added to the mixture with subsequent bath sonication for 1 h with cooling water circulation. Oxidation of the dispersed mixture was progressed by gentle stirring at 50℃ for 12 h. Subsequently, deionized (DI) water (800 mL) was slowly added to the mixture in an ice bath and the reaction was terminated by the addition of hydrogen peroxide (H_2_O_2_) (3 mL). The mixture was vacuum-filtered through a 0.2 μm hydrophilic PTFE membrane filter, after which the compact filtered cake was washed twice with DI water (200 mL), hydrochloric acid (HCl) (200 mL), and ethanol (200 mL). Finally, the vacuum filtrate was washed with diethyl ether (200 mL) and dried overnight at 80°C in an air circulator.

*The synthesis of nano-onion-acyl chloride*: Oxidized nano-onion-COOH was dispersed in the 100 mL of thionyl chloride and 0.5 mL of anhydrous *N,N*-dimethylformamide (DMF) was slowly added dropwise via a syringe while sonicating in an ice bath for 15 min. After N_2_ purging for 30 min, acyl formation was progressed by stirring at 70℃ and distilling for 24 h. The resulting nano-onion-acyl chloride cake was filtered through a 0.2 μm hydrophilic PTFE membrane and residual thionyl chloride was removed by washing 5 times with anhydrous tetrahydrofuran (THF).

*The synthesis and amination of MoS*_2_* nanosheets*: Powdered pristine MoS_2_ (120 mg) was added to *N*-vinyl-2-pyrrolidone (NVP) (60 mL) and dispersed into smaller sheets via probe sonication at 10℃ for 8 h. Large particles were removed by centrifugation at 4000 rpm for 5 min, after which the supernatant was filtered through a syringe filter (Advantec, HP045AN). The filtered solution was centrifuged at 14600 rpm for 10 min, after which the MoS_2_ nanosheets settled on the bottom were carefully taken. Residual NVP was removed by washing with isopropyl alcohol, followed by drying under vacuum at 40℃. MoS_2_ nanosheets (1 mg) were dispersed in 1 mL of anhydrous DMF by bath sonication. Cysteamine (5 mg) was dissolved in 1 mL of anhydrous DMF, after which cysteamine solution (200 μL) was added to 1 mg/mL of MoS_2_ nanosheets in DMF. The mixture was dispersed via bath sonication for 1 h in an ice bath and then left at room temperature for 24 h. Unreacted cysteamine was removed by washing with DMF, after which the aminated MoS_2_ nanosheets were dried under vacuum at 60°C.

*Formation of the nano-onion/MoS*_2_ *nanosheet composites*: Nano-onion-acyl chloride and MoS_2_ (1 mg/ml) were dispersed in anhydrous DMF via bath sonication after which it was added to the nano-onion-acyl chloride solution dropwise to achieve weight ratios of 1:1 to 1:8 via sonication in an ice bath for 30 min followed by vortex for 24 h.

### Physical characterization

The crystalline structures of pristine MoS_2_ and MoS_2_ nanosheets were characterized via X-ray diffraction (XRD) (D8-Advance X-ray diffractometer, Bruker Corp., MA, USA) equipped with a Cu Kα radiation source (λ = 0.154 mm; 40 kV; 40 mA) and a high-speed LynxEye detector. XRD spectra of MoS_2_ pristine and MoS_2_ nanosheet were recorded over the 2θ range from 10º to 70º with a step size of 0.02.

UV absorption by the MoS_2_ nanosheets and the 1:1–1:8 nano-onion/MoS_2_ nanosheet composites was measured with a V-670 UV–vis/NIR spectrophotometer (JASCO Corp., Tokyo, Japan) using a synthetic quartz cuvette with a 1 cm light path (Hellma Analytics, Germany). The conditions used were a scanning speed of 200 nm/min, a data interval of 1 nm, UV/vis analysis with a bandwidth of 1.0 nm, and near-infrared (NIR) analysis with a bandwidth of 2.0 nm. Nano-onion/MoS_2_ composites and MoS_2_ nanosheets (0.1 mg/mL DI water, 2 mL) were prepared for UV measurements in the range from 300 to 900 nm.

X-ray photoelectron spectroscopy (XPS) measurements were performed with an Al Kα energy source (ThermoFisher Scientific Co., USA), and the resulting spectra were analyzed by using Avantage software. Fourier-transform infrared spectroscopy (FT-IR) was performed with a Nicolet 6700 (ThermoFisher Scientific CO., USA) with KBr pellets. The morphologies of the MoS_2_ nanosheets and nano-onion/MoS_2_ nanosheet composites with various weight ratios were characterized by using high-resolution transmission electron microscopy (HRTEM, 200 kV, JEM-3010, JEOL, Tokyo, Japan). Before HRTEM visualization, MoS_2_ nanosheets and nano-onion/MoS_2_ nanosheet composites (1 mg) were dispersed in DI water (1 mL) and diluted in ethanol to a concentration of 0.1 mg/ml, which was appropriate for imaging. Dispersions of the composites (10 μl) were doped on lacey Formvar/Carbon with a 200 mesh grid (TED PELLA Co., California, USA) and dried for 10 min at 60℃ in a vacuum oven.

#### Deposition of aminated MoS_2_ nanosheets or nano-onion/MoS_2_ nanosheet composites on indium tin oxide (ITO) substrates

A three-electrode system was prepared using acetone-wiped ITO glass as the working electrode, Pt wire as the counter electrode, and Ag/AgCl as the reference electrode. Aminated MoS_2_ nanosheets or nano-onion/MoS_2_ nanosheet composite dispersions in 0.1 M sodium perchlorate (NaClO_4_·H_2_O; 0.2 mg/ml) employed as the electrolyte. Aminated MoS_2_ nanosheets or a nano-onion/MoS_2_ composite were electrodeposited onto an ITO substrate to provide a DNA-sensing surface. The electrodeposition conditions were a potential range from 0.1 V to − 1.7 V and a scan rate of 0.05 V/s.

#### Preparation of the oligomeric solutions

The following ssDNA sequences (1 μmol) were purchased from Macrogen (Seoul, Korea):

Probe DNA (HPV-16): thiol modified 5'-GAG GAG GAT GAA ATA GAT GGT-3') 24 mer.

Probe DNA (HPV-18): thiol modified 5'-CAC ATT GTG GCA CAA TCT TTT A-3') 22 mer.

Non-complementary DNA: 5'-AAA AAA AAA AAA AAA AAA AAA A-3'.

To provide HPV16-positive DNA in Siha cells and HPV 18-positive DNA in Hela cells, solutions of the appropriate ssDNA were prepared in 0.1 M Tris buffer (pH 7.0) to a final concentration of measurement.

#### DNA hybridization

Aminated MoS_2_ nanosheets or a nano-onion/MoS_2_ nanosheet composite electrodeposited onto an ITO substrate for 24 h at 4°C were exposed to an HPV16 or HPV18 probe DNA solution (10 μM, Tris pH 7.0) and immobilized via chemisorption. Electrodeposited plate electrode of No/MoS_2_ nanosheet and MoS_2_ nanosheet was placed into the plate material evaluating cell (Labsolutions, SP) for each electrode. Under wet condition, thiolated DNA probe chemisorption, washing, target DNA hybridization, and buffer changing etc. were carried out in a subsequent mode until DPV measurement was completed. The plate material evaluating cell had a 7.8 mm diameter liquid contact area, was filled with 1 mL electrolytes solution, and DNA concentration was calibrated based on the 1 mL evaluating cell volume. Hybridization was performed by exposing the electrode surface to a solution of HPV positive Siha or Hela target DNA along with MB as a redox indicator in 1 μM in KCl (pH 7.0) for 15 min at room temperature. Nonhybridized DNA was removed with same buffer. Schematic process of immobilization and hybridization of DNA was illustrated for HPV sensing nano-onion /MoS_2_ nanocomposite platform with electrochemical detection (Scheme 2).

#### Electrochemical characterization

The electrical characteristics of the nano-onion/MoS_2_ nanosheet composite-covered electrode were measured via cyclic voltammetry (CV) while the dsDNA sensing via the intercalated MB was characterized by using DPV. To prepare the electrode, 0.5% Nafion solution in water was mixed with a nano-onion/MoS_2_ composite (1 mg/mL). The mixture was sonicated with a power amplitude of 30% at 5℃ for 10 min to disperse it. A drop (10 μL) of the dispersion was placed on the working electrode of glassy carbon (3 mm in I.D.) and dried at 60℃ for 1 h in an oven. CV measurements of the prepared working electrodes of nano-onion/MoS_2_ composites with various ratios (1:1–1:8) were measured using Ag/AgCl as the reference electrode and Pt as the counter electrode in a solution of 0.1 M potassium hydroxide (KOH). The potentiostat was set at − 0.2 V at 1.0 V and − 0.8 V at 0.2 V and the scan rate was varied as 0.05, 0.1, or 0.5 V/s.

DPV measurements were performed on the 1:1 nano-onion/MoS_2_ nanosheet composite and evaluated in a flow cell with a potentiostat/galvanostat driven by DAEWON software. An ITO electrodes covered with the electroplated nano-onion/MoS_2_ nanosheet composite and MoS_2_ nanosheet electrode were used as the working electrode, PT as the counter electrode, and AG/AgCl as the reference electrode. The three-electrode system for the electrochemical measurements was operated under the following parameter settings: an amplitude of 0.025 V, a modulation time of 0.05 s, an interval time of 0.5 s, sampling time of 0.02 s and a step potential of 0.005 V.

## Supplementary Information

**Additional file 1: Figure S1.** X-ray diffractionspectra of molybdenum disulfidenanosheets and bulk MoS_2_ powder. **Figure S2.** XPS survey scan of NO/MOS_2_ nanosheet composite with 1:1 ratio. **Figure S3.** CV of nanoonionand MoS_2_ nanosheet.

## Data Availability

DPV setting condition was maintained constant for the measurement of Figure 6, 7, 8. So data infomation is sufficient.
