# Supplementary material for: A graphitic nano-onion/molybdenum disulfide nanosheet composite as a platform for HPV-associated cancer-detecting DNA biosensors
Source: J Nanobiotechnology. 2023 Jun 10;21:187. doi: 10.1186/s12951-023-01948-6 (PMC10257840; doi:10.1186/s12951-023-01948-6)
Supplement: Supplementary file 1 — Additional file 1: Figure S1. X-ray diffractionspectra of molybdenum disulfidenanosheets and bulk MoS2 powder. Figure S2. XPS survey scan of NO/MOS2 nanosheet composite with 1:1 ratio. Figure S3. CV of nanoonionand MoS2 nanosheet. [file 12951_2023_1948_MOESM1_ESM.docx]

Supporting Information

A graphitic nano-onion/molybdenum disulfide nanosheet composite as a platform for HPV-associated cancer-detecting DNA biosensors

Youngjun Kim, Eunah Kang

Department of Chemical Engineering and Materials Science

Chung-Ang University


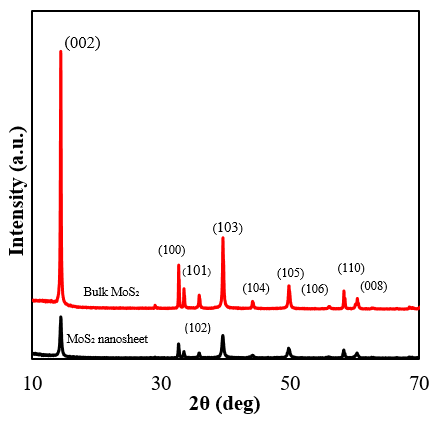


Figure S1. X-ray diffraction (XRD) spectra of molybdenum disulfide (MoS_2_) nanosheets and bulk MoS_2_ powder.

Typical XRD spectra of the bulk MoS₂ used for exfoliation and the subsequently obtained nanosheets are shown in Figure S1. The reflection peaks were assigned to the family lattice planes of bulk MoS₂ (JCPDS card no.77-1716). After probe sonication in N-vinyl-2-pyrrolidone for 8 h, the intensity of the (002) peak decreased dramatically, implying the formation of few-layered MoS₂ nanosheets. No new peaks appeared in the XRD spectrum of the exfoliated MoS₂ nanosheets.

Figure S2. XPS survey scan of NO/MOS_2_ nanosheet composite with 1:1 ratio.


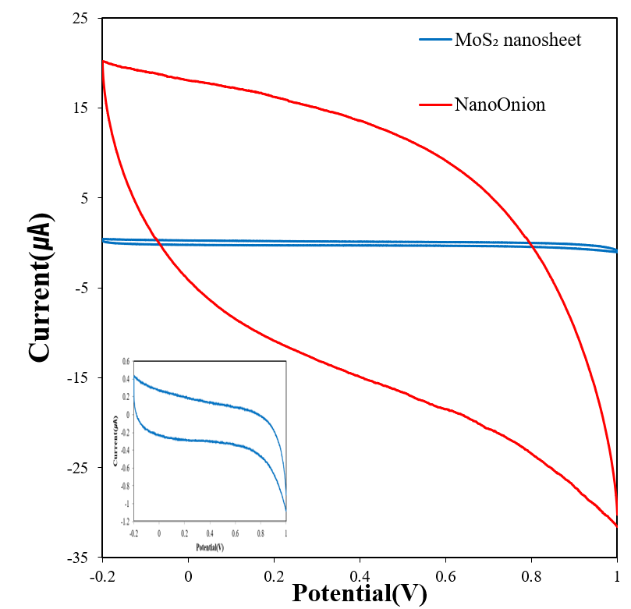


Figure S3. CV of nanoonion (NO) and MoS_2_ nanosheet.
